# Serological detection and analysis of anti-VP1 responses against various enteroviruses (EV) (*EV-A*, *EV-B* and *EV-C*) in Chinese individuals

**DOI:** 10.1038/srep21979

**Published:** 2016-02-26

**Authors:** Caixia Gao, Yingying Ding, Peng Zhou, Jiaojiao Feng, Baohua Qian, Ziyu Lin, Lili Wang, Jinhong Wang, Chunyan Zhao, Xiangyu Li, Mingmei Cao, Heng Peng, Bing Rui, Wei Pan

**Affiliations:** 1 Department of Medical Microbiology and Parasitology, School of Basic Medicine, Second Military Medical University; 2 Department of Physiology, Anhui Medical University, Hefei, China; 3 Department of Blood Transfusion, Changhai Hospital, Second Military Medical University, Shanghai, China

## Abstract

The overall serological prevalence of EV infections based on ELISA remains unknown. In the present study, the antibody responses against VP1 of the *EV-A* species (enterovirus 71 (EV71), Coxsackievirus A16 (CA16), Coxsackievirus A5 (CA5) and Coxsackievirus A6 (CA6)), of the *EV-B* species (Coxsackievirus B3 (CB3)), and of the *EV-C* species (Poliovirus 1 (PV1)) were detected and analyzed by a NEIBM (novel evolved immunoglobulin-binding molecule)-based ELISA in Shanghai blood donors. The serological prevalence of anti-CB3 VP1 antibodies was demonstrated to show the highest level, with anti-PV1 VP1 antibodies at the second highest level, and anti-CA5, CA6, CA16 and EV71 VP1 antibodies at a comparatively low level. All reactions were significantly correlated at different levels, which were approximately proportional to their sequence similarities. Antibody responses against EV71 VP1 showed obvious differences with responses against other *EV-A* viruses. Obvious differences in antibody responses between August 2013 and May 2014 were revealed. These findings are the first to describe the detailed information of the serological prevalence of human antibody responses against the VP1 of *EV-A, B* and *C* viruses, and could be helpful for understanding of the ubiquity of EV infections and for identifying an effective approach for seroepidemiological surveillance based on ELISA.

An increasing number of enteroviruses (EV), a genus of the *Picornaviridae* family, continue to be identified, and currently these viruses are phylogenetically divided into 12 species: enterovirus A, B, C, D, E, F, G, H and J (*EV A–J*), and rhinovirus A, B and C. Apart from the three rhinovirus species, four (*EV-A, B, C* and *D*) of the enterovirus species infect humans. *EV-A* consists of 25 types including CA, EV71, and simian (or baboon) enteroviruses. *EV-B* consists of 63 serotypes including CB, CA9, echoviruses (E), and simian enteroviruses. *EV-C* consists of 23 types including three PV, CA, and *EV-C* viruses. *EV-D* consists of five types including chimpanzee and gorilla viruses^1^,^2^ ([ http://www.picornaviridae.com], September, 2015).

The vast majority of enteroviruses (*EV-A, B, C* and *D*) infect the human gastrointestinal tract and can spread to other organs, such as the heart or the central nervous system. Most EV infections are asymptomatic, but over 20 clinically recognized syndromes, including poliomyelitis, encephalitis, meningitis, HFMD, myocarditis, respiratory illnesses, febrile illnesses, pleurodynia, herpangina, conjunctivitis, gastroenteritis, myopericarditis, hepatitis, and pancreatitis, have been frequently associated with many of the 103 EV types^2^. Among these diseases, HFMD has been the most common EV disease that usually affects children, particularly those less than 5 years old^3^,^4^,^5^. Several large outbreaks of HFMD have been reported in eastern and southeastern Asian countries and regions during the late 20th century^6^,^7^,^8^,^9^. Since 2008, a dramatic increase in HFMD prevalence has been reported in mainland China^3^,^10^,^11^,^12^. The HFMD etiological agents usually consist of viruses in *EV-A* and *EV-B*, and most of these include CA2-8, 10, 12, 14, 16 and EV71, which are *EV-A* viruses^13^,^14^. CA16 and EV71 are major etiological agents for HFMD.

EV virions are comprised of 60 copies of four capsid proteins (VP1, VP2, VP3 and VP4) that form a symmetrical icosahedral structure. The capsid proteins VP1, VP2 and VP3 are exposed on the virus surface, and the smallest protein, VP4, is arranged inside the icosahedral lattice^15^,^16^,^17^,^18^. The VP1 protein is highly exposed and has been suggested to play an important role in viral pathogenesis and virulence^19^,^20^,^21^. The viral structural proteins VP1, VP2, VP3 are highly structured, comprising a beta sheet, and are likely the principal targets for the host’s humoral immunity responses^16^,^18^,^19^,^22^,^23^,^24^. The neutralizing antibody assay has been the only effective serological assay to successfully assess human EV epidemics^25^,^26^,^27^,^28^. However, this technique has provided little cumulative evidence to evaluate the overall serological prevalence levels of various EV types and species. Our previous study, through the use of a NEIBM-based ELISA, demonstrated that the non-neutralizing antibody responses against EV71 were predominantly in response to VP1^29^. In this present study, the VP1 proteins of the major etiological agents for HFMD in *EV-A* (EV71, CA16, CA5 and CA6) and in *EV-B* (CB3) and for poliomyelitis in *EV-C* (PV1) were prepared and used to detect and analyze the anti-VP1 antibody responses by a NEIBM-based ELISA and a competitive ELISA in Shanghai blood donors in August 2013 and May 2014. Different and related antibody responses against VP1 from *EV-A, B* and *C* were revealed, which demonstrated different levels of serological prevalence among the various EV types and species. These results could be helpful for understanding ubiquitous EV infections and the establishment of EV seroepidemiological surveillance based on a convenient ELISA.

## Results

### Production of various recombinant VP1 proteins

**Figure 1:**
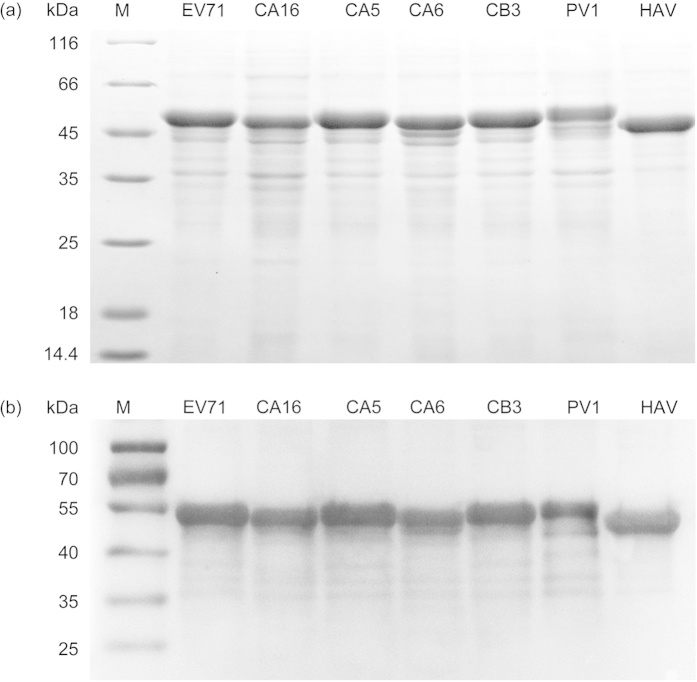
Expression and purification of the recombinant EV71, CA16, CA5, CA6, CB3, PV1 and HAV VP1 proteins. (**a**) Purified recombinant VP1 proteins were separated on 12% SDS-PAGE gel and stained with Coomassie blue. The expression of the recombinant EV71, CA16, CA5, CA6, CB3, PV1 and HAV VP1 proteins was induced with IPTG. The relative molecular weights (MW) of the expressed products were 50.37 kDa for EV71, 50.38 kDa for CA16, 50.20 kDa for CA5, 50.86 kDa for CA6, 50.45 kDa for CB3, 51.16 kDa for PV1 and 48.08 kDa for HAV. The expressed products were purified using Ni-NTA column affinity chromatography. (**b**) Western blot analysis of the expression of His-probe recombinant EV71, CA16, CA5, CA6, CB3, PV1 and HAV VP1 proteins.

In our previous study, an anti-VP1 response was found to be the predominant antibody response against EV71 capsid proteins^29^. In this present study, the VP1 of various viruses from three species of the *Enterovirus* genus, EV71, CA16, CA5 and CA6 from *EV-A*, CB3 from *EV-B*, PV1 from *EV-C*, and HAV from a *Hepatovirus* genus virus (as a control) were expressed as fusion proteins in E. *coli* and verified by SDS-PAGE (Fig. 1a). The size of each recombinant protein was in agreement with the expected molecular weight. All of the fusion proteins were found in inclusion bodies. Although these proteins were insoluble, they could be easily solubilized in 8 M urea and conveniently purified under denaturing conditions. Western blot analysis confirmed the expression of the purified proteins (Fig. 1b).

### Antibody responses against VP1 of *EV-A*, *EV-B* and *EV-C* demonstrated different prevalence levels and high correlations

**Figure 2:**
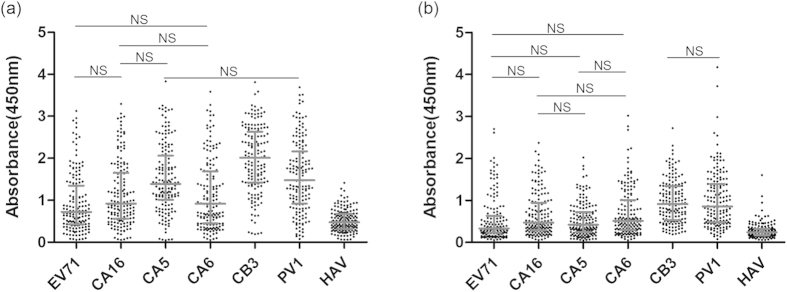
Comparison of the antibody reactivity against VP1 of EV71, CA16, CA5, CA6, CB3, PV1 and HAV in serum samples in August 2013 (**a**) and May 2014 (**b**). Each symbol represents an individual sample, and the lines indicate X_25%_, X_50%_ and X_75%_ value of the group from the bottom to the top, respectively. “NS” represents no significant difference between the two groups. The differences were significantly different between the other groups.

To characterize the antibody reactions against VP1 of the various viruses, a NEIBM-based ELISA was performed to determine the sample reactivities against VP1 of EV71, CA16, CA5 and CA6 from *EV-A*, CB3 from *EV-B* and PV1 from *EV-C* in August 2013 and May 2014. As shown in Fig. 2a, the anti-VP1 reactivities of the samples from August 2013 demonstrated four reactivity levels. The highest level was against CB3 VP1, which was significantly stronger than that against PV1 VP1, HAV VP1 and the VP1 of EV71, CA16, CA5 and CA6. The second highest level was against PV1 VP1, which was significantly stronger than against HAV VP1 and the VP1 of EV71, CA16 and CA6. A low level was observed against the VP1 of the *EV-A* members EV71, CA16, CA5 and CA6, which was only significantly stronger than that against HAV VP1, while the lowest level observed was against HAV VP1. Moreover, the anti-VP1 responses against *EV-A* members EV71, CA16, CA5 and CA6 also demonstrated different reactivity levels, with the anti-CA5 VP1 response significantly stronger than that of the anti-EV71 VP1 and the anti-CA6 VP1 responses. Similar to these results from August 2013, the anti-VP1 reactivities from the May 2014 samples demonstrated three response levels (Fig. 2b): the highest level was against CB3 VP1 and PV1 VP1, the second highest level was against *EV-A* VP1, and the lowest level was against HAV VP1. However, the anti-VP1 reactivities against the different *EV-A* viruses demonstrated the same levels, which was different from what was observed in the samples from August 2013. These results could suggest different levels of serological prevalence of various EV infections. In addition, the significantly higher sample reactivity against the VP1 of CB3 or CA5 than against the VP1 of PV1 or EV71, respectively, was found in the samples from August 2013 but not in the samples from May 2014 (Fig. 2).

**Figure 3:**
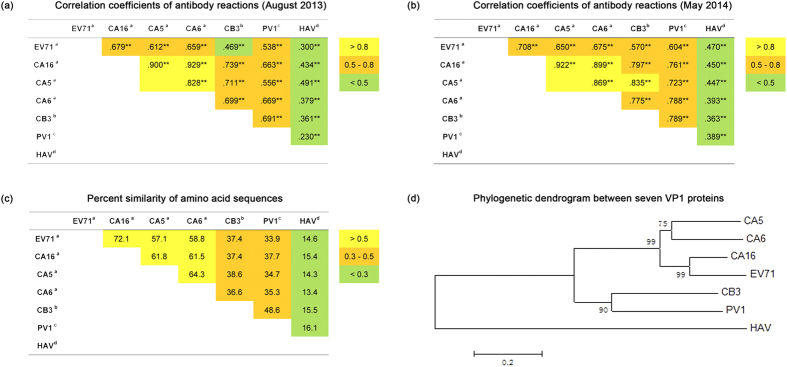
Correlation coefficients of antibody reactions, percent similarity of amino acid sequences and phylogenetic dendrogram between seven VP1 proteins. **represents p < 0.01. ^a^Enterovirus A. ^b^Enterovirus B. ^C^Enterovirus C. ^d^Hepatitis A virus. The phylogenetic dendrogram were drawn on the basis of the amino acid sequences of the entire VP1 protein using the Neighbor-Joining method of MEGA software, and the amino acid sequences of EV71 VP1, CA16 VP1, CA5 VP1, CA6 VP1, CB3 VP1, PV1 VP1 and HAV VP1 were obtained from GenBank (GenBank accession number: EU703812, ACT52617.1, AEJ54594.1, AGI41373.1, AGR84785.1, AGE13930.1 and A3FMB2.1, respectively).

To further characterize the antibody reactions against these enteroviruses, a correlation analysis of the antibody reactions was performed. As shown in Fig. 3, the reactions against VP1 for all of the detected viruses showed significant correlations but at different correlation levels. The correlations between reactions against VP1 of the various *EV-A* viruses, except for EV71, were the highest, with correlation coefficients above 0.8 at both the August 2013 and May 2014 time points (Fig. 3a,b). The correlations between reactions against VP1 of EV71 and that of other *EV-A* viruses were the second highest, with correlation coefficients between 0.612 and 0.708. The correlations between reactions against the VP1 of viruses in various EV species (EV71, CA16, CA5 and CA6 in *EV-A*, CB3 in *EV-B* and PV1 in *EV-C)* were also generally the second highest, with correlation coefficients between 0.5 and 0.8 with two exceptions: the correlation between reactions against EV71 VP1 and CB3 VP1 in August 2013 had a correlation coefficient of 0.469, and the correlation between reactions against CA5 VP1 and CB3 VP1 in May 2014 had a correlation coefficient of 0.835. The correlations between reactions against the VP1 of *Enterovirus* genus viruses and the VP1 of HAV from the *Hepatovirus* genus were the lowest, with correlation coefficients of less than 0.5. Very interestingly, these results revealed that the correlation levels between the reactions against VP1 of the various viruses were roughly proportional to their sequence similarities, with the exception of EV71 (Fig. 3). These results suggest that the homologous amino acids sequences contribute to the highly significant correlations between the antibody responses against VP1 of the various enteroviruses.

### Obvious differences between the anti-VP1 reactions were revealed by inhibition analysis

**Figure 4:**
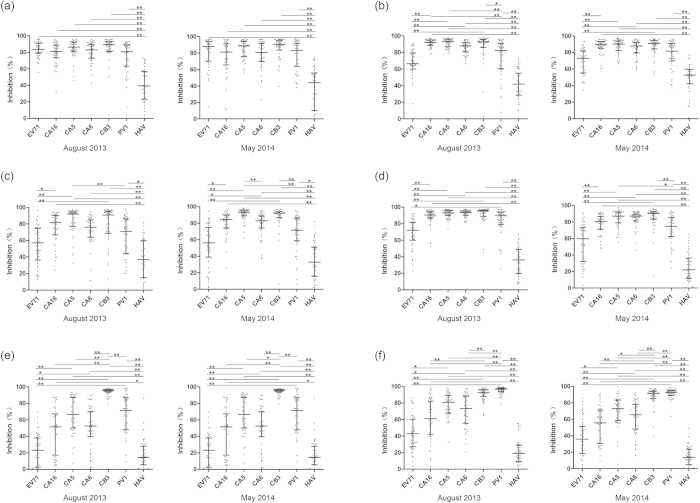
Comparison of inhibition activities to anti-VP1 reactions of EV71 (**a**), CA16 **(b**), CA5 (**c**), CA6 (**d**), CB3 (**e**) and PV1 (**f**) VP1 by seven VP1 proteins, EV71, CA16, CA5, CA6, CB3, PV1 and HAV in August 2013 and May 2014. The percent inhibition from the competitive ELISA was plotted on the y-axis with the seven inhibitor proteins on the x-axis: VP1 of EV71, CA16, CA5, CA6, CB3, PV1 and HAV. The lines indicate X_25%_, X_50%_ and X_75%_ value of the group from the bottom to the top, respectively. Statistical significance was tested using the Nemenyi non-parametric test. *represents p < 0.05, **represents p < 0.01.

To further characterize the antibody reactions in detail, each of the 48 samples that were strongly reactive against VP1 of the various viruses (the serum samples were sorted by the OD value of the anti-VP1 reactivity, and the ones with highest reactivities were chosen) was analyzed in a competitive inhibition ELISA. The results are shown in Fig. 4. The anti-CB3 VP1 samples were completely inhibited by CB3 VP1, partially inhibited by VP1 of PV1 and the *EV-A* viruses, except for EV71, and poorly inhibited by VP1 of HAV and EV71 (Fig. 4e). The anti-PV1 VP1 samples were completely inhibited by the VP1 of both PV1 and CB3, strongly inhibited by the VP1 of CA5, CA6 and CA16, partially inhibited by EV71 VP1, and poorly inhibited by HAV VP1 (Fig. 4f). The samples that were anti-VP1 for CA16, CA5 and CA6 were strongly inhibited by the VP1 of CA16, CA5, CA6, CB3 and PV1, partially inhibited by EV71 VP1, and poorly inhibited by HAV VP1 (Fig. 4b–d). The anti-EV71 VP1 samples were strongly inhibited by the VP1 of all of the viruses except for HAV (Fig. 4a). Moreover, the inhibition of the reactions against VP1 of other viruses in the *Enterovirus* genus or even of the *EV-A* EV71 VP1 by EV71 VP1 was significantly weaker than that by VP1 of other viruses in the *Enterovirus* genus, suggesting a relatively low prevalence level of anti-EV71 VP1. Consistent with the results of the sample reactivities, these results also demonstrated different serological prevalence levels of the various EV infections: the highest level was against CB3 VP1, the second highest level was against PV1 VP1, and a comparatively low level against the VP1 of EV71, CA16, CA5 and CA6 in *EV-A*. In addition, the inhibition against the anti-CA16 VP1, anti-CA5 VP1, or anti-CA6 VP1 samples by the VP1 of some enteroviruses was significantly different in the samples from August 2013 but not in the May 2014 samples (Fig. 4, Table S1).

Correlation analyses of the inhibition of the various anti-VP1 reactions were performed to further characterize the antibody reactions against the VP1 of the various enteroviruses. As shown in Table 1 and Fig. S1, the inhibition of the anti-CB3 VP1 reaction by CB3 VP1 and the VP1 of other viruses did not show a significant correlation, supporting the highest prevalence level of specific antibody responses. The inhibition of anti-PV1 VP1 reactions by PV1 VP1 and the VP1 of other viruses, except for CB3, also did not correlate significantly, while the inhibition of anti-PV1 VP1 reactions by PV1 VP1 and CB3 VP1 showed high correlations, with correlation coefficients of 0.508 and 0.427 in the samples from August 2013 and May 2014, respectively, consistent with the second highest prevalence level of antibody responses specific for PV1 VP1 and cross-reactive for CB3 VP1. The inhibition of reactions that were anti-VP1 for *EV-A* viruses by the VP1 of *EV-A* viruses, except for EV71, showed strong correlations, with correlation coefficients above 0.607 in the samples from August 2013 and May 2014. Moreover, the inhibition of reactions that were anti-VP1 for *EV-A* viruses by the VP1 of viruses in *EV-A,* except for EV71, and by VP1 of CB3 or PV1 also showed strong or significant correlations, with correlation coefficients above 0.620 in many cases. These results suggest a low prevalence level of cross-reactive antibody responses. Intriguingly, the inhibition of reactions against the VP1 of *EV-A* viruses, except for EV71, by EV71 VP1 and by the VP1 of *EV-A* viruses showed significant correlations in only half of the cases, suggesting a different antigenicity for EV71 VP1. In the control, the inhibition of reactions against VP1 of viruses in the *Enterovirus* genus by HAV VP1 in the *Hepatovirus* genus and by VP1 of viruses in the *Enterovirus* genus generally did not significantly correlate. These results could reflect the different antibody responses against CB3 VP1, PV1 VP1, and the VP1 of *EV-A* viruses.

The obvious differences between the antibody responses in August 2013 and May 2014 were also clearly revealed in the analysis of correlations between the inhibition of reactions to anti-VP1 (Table 1, Fig. S1). The correlation between the inhibition of anti-EV71 VP1 reactions by VP1 of EV71 and CB3 in the samples from August 2013 and May 2014 also showed obvious differences, with correlation coefficients of 0.536 and 0.929, respectively. Moreover, the correlation coefficients between the inhibition of anti-CA16 VP1 reactions by VP1 of EV71 and CA16, the inhibition of anti-CA5 VP1 reactions by VP1 of EV71 and CA5, and the inhibition of anti-CA6 VP1 reactions by VP1 of EV71 and CA6 in the August 2013 samples showed significant differences with those of the May 2014 samples, respectively. These results suggested different antibody responses against EV71 VP1 between the samples from August 2013 and May 2014. Such obvious differences were also observed in correlations between the inhibition of anti-CB3 VP1 reactions by VP1 of ether CB3 and PV1 or CB3 and CA6, and the inhibition of anti-CA6 VP1 reactions by VP1 of CA6 and CB3, CA6 and PV1, or CA6 and EV71 (Table 1, Fig. S1), suggesting different antibody responses against PV1 and CA6, respectively.

## Discussion

Information on the prevalence of EV infection has been now gained from a neutralizing antibody assay, but this assay is limited for use as it is time consuming and difficult for large scale detection, requires the use of viable viruses, specialized equipment and trained personnel, and cannot provide information on the levels of prevalence for the various viruses compared to one another. Our previous study, using a NEIBM-based ELISA method, revealed that the seroprevalence and the reactivity of anti-EV71 VP1 antibody response were significantly higher and stronger than that of the antibody response anti-EV71 VP0 and anti-EV71 VP3 in both normal adults and severe HFMD cases, demonstrating that the non-neutralizing antibody responses against EV71 were predominantly in response to VP1^29^. The studies on structure of EV71 and CA16 also support VP1 is the principle capsid antigen^18^,^24^. In this present study, the combined detection and analysis of the anti-VP1 proteins of various enteroviruses by a NEIBM-based ELISA and a competitive ELISA clearly demonstrated that the prevalence of anti-CB3 antibodies from *EV-B* were at the highest level, followed by anti-PV1 antibodies from *EV-C* at the second highest level, anti- EV71, CA16, CA5 and CA6 antibodies from *EV-A* at a comparatively low level, and anti-HAV antibodies at the lowest level (Figs 2 and 4). In theory, the highest level of sample reactivity against CB3 VP1 indicated the strongest antibody response against CB3, which could be comprised of both specific antibodies elicited by a CB3 infection and cross-reactive antibodies elicited by infections with other enteroviruses. In this study, the complete inhibition of anti-CB3 VP1 reactions by only CB3 VP1 (Fig. 4e) and the lack of correlation between the inhibition of anti-CB3 VP1 reactions by CB3 VP1 and by the VP1 of the other detected viruses (Table 1, Fig. S1) demonstrated that the antibody response against CB3 VP1 was mainly attributable to the specific antibodies elicited by CB3 infection. Consistent with the second highest level of sample reactivity against anti-PV1 VP1, complete inhibition of the reaction by only PV1 VP1 and CB3 VP1 (Fig. 4f) and a significant correlation only between inhibition of the reaction by PV1 VP1 and CB3 VP1 (Table 1, Fig. S1) demonstrated that the detected antibody response against PV1 VP1 was mainly attributable to both the specific antibodies elicited by the PV1 infection and cross-reactive antibodies mainly elicited by CB3 infection. With a comparatively low level of sample reactivity against VP1 of EV71, CA16, CA5 and CA6 in *EV-A* (Fig. 2), the strong inhibition of the reactions by the VP1 of *EV-A* viruses and of PV1 and CB3 (Fig. 4a–d) and a highly significant correlation between the inhibition of the reactions by the VP1 of other *EV-A* viruses and of CB3 and PV1 (Table 1, Fig. S1) demonstrated that these antibody responses were mainly attributable to cross-reactive antibodies elicited by the infections of other *EV-A* viruses and of PV1 and CB3, with little contribution from specific antibodies. As a control, the anti-HAV VP1 reactions occurred at the lowest level (Fig. 2) and weakly correlated with anti-VP1 reactions for various enteroviruses (Fig. 3). It is interesting to note that the prevalence levels of the reactions for anti-CB3 in *EV-B*, anti-PV1 in *EV-C* and anti-VP1 in *EV-A* were generally proportional to the numbers of isolated types or serotypes in these three species: 63 serotypes in *EV-B*, 23 types in *EV-C*, and 25 types in *EV-A*^2^ ([ http://www.picornaviridae.com], September, 2015). The regular vaccination with the attenuated live polio vaccine in China could well explain the second highest level of prevalence of anti-PV1 responses in the present study. The anti-HAV VP1 reactions occurred at the lowest level, and the explanations were as follows. Firstly, as a genus of the *Picornaviridae* family, only one serotype of HAV has been found, and there are no cross-reactive antibodies which could contribute to anti-HAV VP1 level. Secondly, unlike OPV, the live attenuated HAV vaccine was administered subcutaneously, could not generate the transmissible infection in the human population, and thus contribute little to the increase of anti-HAV VP1 level. Thirdly, as EV71, CA16, CA5, CA6, CB3 and PV1 are members of *Enterovirus* genus, and HAV is a member of *Hepatovirus* genus, the different transmission capability between them may be the most reasonable cause of lower level of anti-HAV VP1 compared with that of EVs. These results are the first to describe a rough approximation of the serological prevalence levels of three enterovirus species that infect the human gastrointestinal tract, demonstrating the highest prevalence level of infection with CB3, the major etiologic agent of myocarditis^30^,^31^,^32^ and a comparatively low prevalence level of infection with EV71 and CA16, the two major etiologic agents of HFMD, providing new insights into ubiquitous EV infections.

Very interestingly, the reactions against the various detected viruses, except for EV71, in both the *Enterovirus* genus and the *Hepatovirus* genus were significantly correlated to different levels that are basically proportional to their sequence similarities (Fig. 3), exhibiting the nature of sequence-based cross-reactiveness of antibodies in response to infections by enteroviruses. The antibodies elicited by the VP1 of one virus could react with its own VP1 and cross-react with the VP1 of other viruses with homologous sequences at the degree proportional to the level of their sequence similarities. This finding demonstrated that the high sequence similarity of enteroviruses has a significant impact on antigenicity. What its significance may be for the viral immunology remains unknown.

However, correlations between the inhibitions of the reactions seemed to suggest something different. Although the correlations between the inhibitions of anti-VP1 of EV-A virus reactions by the VP1 of *EV-A* viruses, except for EV71, were the highest, the correlations between the inhibition of anti-VP1 reactions of these viruses by VP1 of CB3 or PV1 and by VP1 of *EV-A* viruses were similarly high or even higher in many cases (Table 1, Fig. S1). This might be because the VP1 of CB3 or PV1 could effectively inhibit the reactions of high levels of cross-reactive antibodies elicited by the infections of CB3 or PV1 with the VP1 of *EV-A* viruses. In contrast, the anti-CB3 VP1 reactions were poorly inhibited by the VP1 of other enteroviruses, and the inhibition of anti-CB3 reactions by VP1 of CB3 and by VP1 of other enteroviruses did not significantly correlate (Table 1, Fig. S1), possibly indicating the anti-CB3 antibodies reacted with CB3 VP1 as specific antibodies and with VP1 of other enteroviruses as cross-reactive antibodies in different ways. The inhibition of anti-PV1 VP1 reactions, which was at the second highest prevalence level, by PV1 VP1 significantly correlated only with inhibition by CB3 VP1 but not by the VP1 of *EV-A* viruses and HAV (Table 1, Fig. S1), suggesting a similar explanation. These findings indicated that the inhibition assay and analysis could distinguish between specific antibody responses and cross-reactive antibody responses.

Very interestingly, the antibody responses against EV71 VP1 showed obvious differences with that of responses against other *EV-A* viruses. The correlations between anti-EV71 VP1 responses and anti-VP1 responses against other *EV-A* viruses were low compared to that between anti-VP1 responses against other *EV-A* viruses to each other and were not proportional to the sequence similarities (Fig. 3), indicating that mutations in EV71 VP1 have a particular impact on its antigenicity. Moreover, EV71 VP1 showed a significantly lower level of inhibition potency against anti-VP1 responses of other *EV-A* viruses, CB3 and PV1, compared with that of other *EV-A* viruses (Fig. 4). Consistently, the correlations between inhibition of reactions against anti-VP1 of other *EV-A* viruses by EV71 VP1 and by VP1 of other *EV-A* viruses were also obviously lower compared with that by VP1 of other *EV-A* viruses to each other (Table 1, Fig. S1). These findings may suggest that the antigenicity of EV71 VP1 is distinct from that of other *EV-A* viruses. What its significance may be for viral immunity and pathogenesis remains of interest.

Many obvious differences could be found in the pattern of sample reactivities, the pattern of inhibition, and the correlations between inhibition in the samples from August 2013 and May 2014. The significantly higher sample reactivity against VP1 of CB3 or CA5 than that against VP1 of PV1 or EV71, respectively, was found in the samples from August 2013 but not in those from May 2014 (Fig. 2). The inhibition against anti-CA16 VP1 reactions by CB3 and PV1, by the CA5 and PV1, and by the CA16 and PV1 showed significant differences, respectively, in the samples from August 2013 but not in those from May 2014 (Fig. 4, Table S1). The inhibition levels against anti-CA5 VP1 reactions by CA5 and CA6 or by the CB3 and PV1, as well as by the CA6 and EV71, showed significant differences, respectively, in the samples from May 2014 but not in those from August 2013 (Fig. 4, Table S1). The inhibition against anti-CA6 VP1 reactions by CB3 and PV1 or by CA5 and PV1 showed significant differences, respectively, in the samples from May 2014 but not in those from August 2013, and that by EV71 and PV1 showed significant differences in the samples from August 2013 but not from those in May 2014 (Fig. 4, Table S1). However, these obvious differences in the pattern of sample reactivities and the pattern of inhibition provided little useful information to help us understand and evaluate the prevalence of infections by enteroviruses. Intriguingly, some obvious differences in correlations between the inhibition of reactions concerning EV71 VP1 in the August 2013 and May 2014 samples (Table 1) seemed to provide useful information. The correlation coefficient of inhibition of the anti-EV71 VP1 reactions by EV71 and by CB3 was 0.929 in the May 2014 samples, compared with 0.536 in the August 2013 samples. Considering the highest prevalence level of the anti-CB3 VP1 reactions, the antibodies against EV71 VP1 in the May 2014 samples should be comprised of only antibodies produced by CB3 infection that react with EV71 VP1 and CB3 VP1 in same way, and could be effectively inhibited by both EV71 VP1 and CB3 VP1, producing the correlation coefficient of 0.929, while antibodies against anti-EV71 VP1 in the August 2013 samples could be comprised of either antibodies produced by CB3 infection or antibodies produced by EV71 infection that react with EV71 VP1 and CB3 VP1 in some different way, producing the low correlation coefficient of 0.536, which could suggest an epidemic of EV71 infection. Consistently, the correlation coefficients between the inhibition of anti-CA16 VP1 reactions by EV71 VP1 and CA16 VP1 and the inhibition of anti-CA5 VP1 reactions by EV71 VP1 and CA5 VP1 in May 2014 were 0.378 and 0.431, respectively, indicating a significant correlation, while these correlation coefficients in August 2013 were 0.194 and −0.041, respectively, indicating no significant correlation. The addition of the antibodies against EV71 VP1 to the antibodies against CB3 VP1 could also be responsible for the reduction of the correlation coefficients between the inhibition of the anti-CA16 VP1 reactions by EV71 VP1 and CA16 VP1 and the inhibition of anti-CA5 VP1 reactions by EV71 VP1 and CA5 VP1 in August 2013. Perhaps this dynamic change in correlation coefficients between the inhibition of anti-EV71 VP1 reactions by EV71 and CB3 at different times could be useful for the evaluation of an EV71 epidemic. It is of great interest to verify this speculation.

This study represents the first serological detection and analysis of anti-VP1 of *EV-A*, *EV-B* and *EV-C* viruses in Chinese individuals by the combination of a NEIBM-based ELISA and a competitive ELISA assay, which demonstrated the different serological prevalence levels of various enteroviruses, revealed the distinct antigenicity of EV71 VP1, and found obvious differences in antibody responses against the detected enteroviruses at different times. These findings could be helpful for understanding ubiquitous enterovirus infections and identifying an effective approach for seroepidemiological surveillance based on the combination of a NEIBM-based ELISA and a competitive ELISA assay.

## Materials and Methods

### Ethical statement

This study was approved by the Ethics Committee of Changhai Hospital, Shanghai, China. All experiments were performed in accordance with the approved guidelines of the Ethics Committee of Changhai Hospital and the Second Military Medical University. Written informed consent was obtained from all participants in the study.

### Clinical samples

One hundred and fifty-five serum specimens were collected from healthy blood donors at Changhai Hospital, Shanghai, China in August 2013, and one hundred and sixty in May 2014. The relevant information for each of the three hundred and fifteen samples was also recorded (Table S2). All samples were stored at −80 °C in 1.5 ml aliquots.

### Vectors, bacterial strains and reagents

The prokaryotic expression plasmid pET32a and two E. *coli* host strains, BL21 (DE3) and Top10, were purchased from Novagen (Darmstadt, Germany). HRP-LD5 consists of HRP conjugated to LD5, which is a novel evolved immunoglobulin-binding molecule (NEIBM) with a characteristic structure of an alternating B3 domain of Finegoldia magna protein Land the D domain of staphylococcal protein A that creates synergistic double binding sites to the VH3 and Vk regions of Fab as well as to IgG Fc^33^. HRP-LD5 shows high binding affinity for IgM, IgG and IgA^34^. HRP-conjugated goat anti-human polyclonal polyvalent immunoglobulins (G, A, and M) (HRP goat anti-human PcAb) were obtained from Sigma (St. Louis, MO, USA). The expression plasmids of EV71 VP1-pET32a were stored in our laboratory.

### Cloning of the VP1 of the CA16, CA5, CA6, CB3, PV1 and HAV gene fragments and construction of the expression plasmids

The amino acidsequences of CA16 VP1 (Coxsackievirus A16 capsid protein VP1), CA5 VP1 (Coxsackievirus A5 capsid protein VP1), CA6 VP1 (Coxsackievirus A6 capsid protein VP1), CB3 VP1 (Coxsackievirus B3 capsid protein VP1), PV1 VP1 (Poliovirus capsid protein VP1) and HAV VP1 (Hepatitis A virus capsid protein VP1) were obtained from GenBank (GenBank accession numbers: ACT52617.1, AEJ54594.1, AGI41373.1, AGR84785.1, AGE13930.1 and A3FMB2.1, respectively). The six representative DNA sequences were synthesized using sequential OE-PCR^35^ and T/A-cloned into the pMD18-T vector (Takara).These constructs were used as templates to amplify CA16 VP1, CA5 VP1, CA6 VP1, CB3 VP1, PV1 VP1 and HAV VP1 using the primer pairs uCA16/dCA16, uCA5/dCA5, uCA6/dCA6, uCB3/dCB3, uPV1/dPV1 and uHAV/dHAV (Table S3), respectively. Additionally, uCA16, dCA6 and uHAV contain *Nco* I restriction sites. dCA16, dCA6, dCB3 and dHAV contain *Xho* I restriction sites. uCA5 and uPV1 contain *Bam*H I restriction sites. dCA5 and dPV1 contain *Sac* I restriction sites. uCB3 contains *Hin*d III restriction site. The PCR products of CA16 VP1, CA5 VP1, CA6 VP1, CB3 VP1, PV1 VP1 and HAV VP1 were inserted into the cloning sites of the pET32a vector under the T7 promoter, and a His-tag was added at the N-terminus of the target to form a fusion protein. These expression plasmids were individually verified by sequencing analysis.

### Expression and purification of recombinant EV71, CA16, CA5, CA6, CB3, PV1 and HAV VP1 proteins

E. *coli* BL21 (DE3) competent cells transformed with EV71, CA16, CA5, CA6, CB3, PV1 and HAV VP1 expression plasmids were cultured in Luria broth (LB) medium supplemented with 100 μg/ml ampicillin (for E. *coli* transformed with pET32a vector) at 37 °C in a shaker at 200 rpm. When the OD 600 of the culture reached 0.6, IPTG was added to a final concentration of 1 mM. After additional incubation for 2–3 h at 37 °C, the bacteria pellets were harvested through centrifugation at 6000 × g for 20 min. After resuspension in PBS, the bacteria pellets were lysed by ultrasonication. Through centrifugation at 11000 × g for 10 min, the inclusion bodies were solubilized in 8 M urea. The proteins were purified using Ni-NTA resin (Qiagen, Hilden, Germany). The targeted proteins were separated by SDS-PAGE and transferred to polyvinylidene difluoride (PVDF) membranes (Bio-Rad). Membranes were blocked overnight at 4 °C in 5% skimmed milk prepared in PBS-Tween 20 and probed with the His-probe antibodies (mouse monoclonal antibodies) (Santa Cruz). Detection was conducted by incubation with horseradish peroxidase (HRP)-coupled goat anti-mouse secondary antibodies (no. 31430) from Pierce Biotechnology (Rockford, IL, USA) and developed using DAB/H_2_O_2_ color development system.

### Indirect ELISA of antibodies against VP1 of various viruses from the *Enterovirus* genus and HAV from the *Hepatovirus* genus

The anti-VP1 of various viruses from three species of *Enterovirus* genus, EV71, CA16, CA5 and CA6 in *EV-A*, CB3 in *EV-B*, PV1 in *EV-C*, and HAV from the *Hepatovirus* genus was detected by ELISA using the NEIBM-derived conjugate HRP-LD5 (NEIBM-based ELISA). The NEIBM-based ELISA assay using the HRP-LD5 as reporter molecule was applied in anti-EV71 VP1 detection and compared with the common ELISA assay using HRP-conjugated goat anti-human polyclonal polyvalent immunoglobulin as reporter molecule, and exhibited an obviously improved detection effect with stronger reactions of relatively high OD value and more clear reactions of background with relatively low OD value^29^. The detection was conducted as previously described^34^,^36^. Briefly, immune assay strips (Nunc, Rochester, NY, USA) were coated with 1.0 μg of EV71, CA16, CA5, CA6, CB3, PV1 and HAV VP1 in 100 mM carbonate buffer (pH 9.6) and incubated at 37 °C for 3 h. The strips were blocked for 2 h at 37 °C with 200 μl of 15% skimmed milk prepared in PBS-Tween 20. Next, 100 μl of a 20-fold dilution of the plasma sample was added to the appropriate wells. The strips were subsequently placed in a 37 °C incubator for 45 min. After washing four times with wash buffer (0.25% Tris base, 0.05% Tween 20), 100 μl of a 2,000-fold dilution of HRP-LD5 (1 mg/ml) was added to the strip and incubated for 45 min at 37 °C. The strips were developed using 3, 3′, 5, 5′-tetramethylbenzidine (TMB) and hydrogen peroxide mixture. The reaction was stopped by the addition of 2 M sulfuric acid, and the absorbance at 450 nm was read using an ELISA Reader (Biotek, Gene Company Limited, USA).

### Competitive inhibition ELISA

To further characterize the antibody reactions against various VP1 in *Enterovirus* genus, a competitive inhibition ELISA was conducted as described^37^,^38^,^39^. Briefly, the 96-well microtiter plates were coated with 1.0 μg of EV71 VP1 protein in 100 mM carbonate buffer (pH 9.6) overnight at 4 °C and then blocked for 2 h at 37 °C with 200 μl of 15% skimmed milk prepared in PBS-Tween 20. Then, 100 μl of a 20-fold dilution of the plasma samples with a high anti-EV71 VP1 antibody response from two groups were first reacted with 2.0 μg of the inhibitor protein (EV71, CA16, CA5, CA6, CB3, PV1 and HAV) for 1 h at 37 °C, then the serum in the presence (test serum) and absence (serum control) of inhibitor proteins was added into EV71 VP1-coated strips and incubated for 45 min at 37 °C. After incubation, the plates were washed four times with wash buffer followed by the addition of 100 μl of a 2,000-fold dilution of HRP-LD5 (1 mg/ml). The plates were incubated for 45 min at 37 °C and then washed. The plates were developed using a 3, 3′, 5, 5′-tetramethylbenzidine and hydrogen peroxide mixture. The reaction was stopped after suitable color development by the addition of 2 M sulfuric acid, and the absorbance at 450 nm was read using an ELISA Reader. Three parallel wells for each test were detected, and the mean of the absorbance from the three wells were used to calculate the percentage of inhibition. The percentage of inhibition (PI) was calculated as follows: PI = [100 − (absorbance value of test serum − absorbance value of background)/(absorbance value of serum control − absorbance value of background) × 100)], where the absorbance of background was obtained in the absence of sample or HRP-LD5. The detection for antibody reactions against VP1 of CA16, CA5, CA6, CB3 and PV1 were conducted as outlined above.

### Statistical analyses

Statistical analyses were performed using SPSS 17.0 and SAS 9.3 software. All experiments were performed in triplicate, and the values obtained from three replicate samples were averaged for each experiment. The statistical significance was tested using the non-parametric test. Differences between measurements were considered significant at p-values less than 0.05.

## Additional Information

**How to cite this article**: Gao, C. *et al.* Serological detection and analysis of anti-VP1 responses against various enteroviruses (EV) (*EV-A*, *EV-B* and *EV-C*) in Chinese individuals. *Sci. Rep.* **6**, 21979; doi: 10.1038/srep21979 (2016).

## Supplementary Material

Supplementary Information

## Figures and Tables

**Table 1:** Correlation analysis of inhibition to the anti-VP1 reactions of six enteroviruses, EV71, CA16, CA5, CA6, CB3 or PV1 by various VP1 proteins.

Inhibition proteins	Coated antigens^#^											
	EV71^a#^		CA16^a#^		CA5^a#^		CA6^a#^		CB3^b#^		PV1^c#^	
	Aug, 2013	May, 2014	Aug, 2013	May, 2014	Aug, 2013	May, 2014	Aug, 2013	May, 2014	Aug, 2013	May, 2014	Aug, 2013	May, 2014
EV71^a^	**—**	**—**	**0.194**	0.378^**^	**−0.041**	0.431^**^	**0.424** ^**^	0.257	**−0.043**	0.138	**0.141**	**−**0.134
CA16^a^	**0.654** ^**^	0.627^**^	**—**	**—**	**0.758** ^**^	0.772^**^	**0.641** ^**^	0.716^**^	**−0.021**	0.098	**0.169**	0.012
CA5^a^	**0.627** ^**^	0.693^**^	**0.725** ^**^	0.782^**^	**—**	**—**	**0.568** ^**^	0.607^**^	**0.153**	0.172	**0.262**	0.112
CA6^a^	**0.766** ^**^	0.677^**^	**0.622** ^**^	0.807^**^	**0.690** ^**^	0.752^**^	**—**	**—**	**−0.161**	0.340*	**0.2**	0.078
CB3^b^	**0.536** ^**^	0.929^**^	**0.620** ^**^	0.738^**^	**0.738** ^**^	0.705^**^	**0.310** ^*^	0.635^**^	**—**	**—**	**0.508** ^**^	0.427^**^
PV1^c^	**0.531** ^**^	0.707^**^	0.484**	0.741^**^	**0.533** ^**^	0.372^**^	**0.321** ^*^	0.468^**^	**−0.008**	0.319^*^	**—**	**—**
HAV^d^	**0.027**	0.23	**−0.176**	0.003	**−.323** ^*^	**−**.302^*^	**0.221**	**−**0.017	**−0.088**	0.162	**0.163**	**−**0.038

*represents p < 0.05. **represents p < 0.01. ^#^These coated antigens were also used as inhibition proteins. ^a^*Enterovirus A*. ^b^*Enterovirus B*. ^c^*Enterovirus C*. ^d^*Hepatitis* A virus.
