# Supplementary material for: Serological detection and analysis of anti-VP1 responses against various enteroviruses (EV) (EV-A, EV-B and EV-C) in Chinese individuals
Source: Sci Rep. 2016 Feb 26;6:21979. doi: 10.1038/srep21979 (PMC4768086; doi:10.1038/srep21979)
Supplement: Supplementary Information [file srep21979-s1.pdf]

Serological detection and analysis of anti-VP1 responses against various enteroviruses (EV) (*EV-A*, *EV-B* and *EV-C*) in Chinese individuals

Caixia Gao[1#], Yingying Ding[1#], Peng Zhou[2], Jiaojiao Feng[1], Baohua Qian[3], Ziyu Lin[2], Lili Wang[1], Jinhong Wang[1], Chunyan Zhao[1], Xiangyu Li[1], Mingmei Cao[1], Heng Peng[1], Bing Rui[1], Wei Pan[1*]

[1]Department of Medical Microbiology and Parasitology, School of Basic Medicine, Second Military Medical University

[2]Department of Physiology, Anhui Medical University, Hefei 230032, China

[3]Department of Blood Transfusion, Changhai Hospital, Second Military Medical University, Shanghai, China

[#]These authors contributed equally to this work.

*Send correspondence to Prof. Wei Pan, Department of Medical Microbiology and Parasitology, School of Basic Medicine, Second Military Medical University, Shanghai 200433, China.

Tel: +86 21 81870989. E-mail: pwpanwei@126.com

Table S1. Significant differences between the inhibition to anti-VP1 reactions of EV71, CA16, CA5, CA6, CB3, and PV1 by VP1 of various enteroviruses and HAV of serum samples from August 2013 and May 2014.

Reactions	Inhibition proteins	Inhibition proteins: EV71[a]		CA16[a]		CA5[a]		CA6[a]		CB3[b]		PV1[c]		HAV[d]	
		August 2013	May 2014	August 2013	May 2014	August 2013	May 2014	August 2013	May 2014	August 2013	May 2014	August 2013	May 2014	August 2013	May 2014
anti-EV71 VP1	EV71[a]													**	**
	CA16[a]													**	**
	CA5[a]													**	**
	CA6[a]													**	**
	CB3[b]													**	**
	PV1[c]													**	**
	HAV[d]														
anti-CA16 VP1	EV71[a]			**	**	**	**	**	**	**	**				
	CA16[a]											**		**	**
	CA5[a]											*		**	**
	CA6[a]													**	**
	CB3[b]											**		**	**
	PV1[c]													**	**
	HAV[d]														
anti-CA5 VP1	EV71[a]			*	*	**	**		*	**	**				
	CA16[a]													**	**
	CA5[a]								**			**	**	**	**
	CA6[a]													**	**
	CB3[b]												**	**	**
	PV1[c]													*	*
	HAV[d]														
anti-CA6 VP1	EV71[a]			**	**	**	**	**	**	**	**	*			
	CA16[a]													**	**
	CA5[a]												*	**	**

	**CA6** [a]									**	**
	**CB3** [b]								**	**	**
	**PV1** [c]									**	**
	**HAV**[d]										
**anti-CB3 VP1**	**EV71** [a]	**	**	*	**	**	**	**	**		
	**CA16** [a]					**	**			*	*
	**CA5** [a]					**	*			**	**
	**CA6** [a]					**	**			**	**
	**CB3** [b]							**	**	**	**
	**PV1** [c]									**	**
	**HAV**[d]										
**anti-PV1 VP1**	**EV71** [a]	*	**	*	*	**	**	**	**		
	**CA16** [a]					**	**	**	**	**	**
	**CA5** [a]						*	**	**	**	**
	**CA6** [a]					**	**	**	**	**	**
	**CB3** [b]									**	**
	**PV1** [c]									**	**
	**HAV**[d]										

* represents $p < 0.05$, ** represents $p < 0.01$. [a]Enterovirus A. [b]Enterovirus B. [C]Enterovirus C. [d]Hepatitis A virus.

Table S2. Baseline characteristics of the study participants

	Blood Donor	Blood Donor
**Number**[a]	155	160
**Time**	Aug, 2013	May, 2014
**Age (yr)**		
Mean ± s.d.[b]	34.0 ± 5.7	34.6 ± 6.9
Range	21-47	19-48
**Sex (%)**		
Male	59.4%	60.6%
Female	40.6%	39.4%

In China, OPV was administered to children through annual mass campaigns during 1965–1977, and OPV coverage increased during the early stage of the Expanded Programme on Immunization (EPI) from 1978 to 1988. Since 1989, OPV routine immunization has been strengthened with the conduction of supplementary immunization activities (SIAs) and the establishment of AFP surveillance system[1]. Healthy Chinese infants and children received a 3-dose primary series of OPV at 2, 3, 4 months of age, and one-dose OPV at 4 years of age in OPV routine immunization.

A live attenuated hepatitis A vaccine was applied in preventing of the disease in 1992, large scale used in vaccination program in 1995, and incorporated in the EPI in 2008 in China. Chinese children usually received each 1 dose of the live attenuated vaccine or inactivated vaccine subcutaneously at 18 months of age, and 2 years of age in Chinese national immunization program[2,3].

Table S3. Primers for amplifying CA16, CA5, CA6, CB3, PV1 and HAV VP1.

Primers	Sequences (5'- 3')	Description
uCA16	GCCGCGCCATGGCTGGTGACCCGATCGCTGAC	The primer pairs uCA16/dCA16, uCB3/dCB3 and uHAV/dHAV were used to amplify the VP1 of CA16, CB3 and HAV, respectively. uCA16, dCA6, uHAV contain *Nco* I restriction sites (underlined), dCA16, dCA6, dHAV contain *Xho* I restriction sites (underlined).
dCA16	GCCGGCCTCGAGCTACAGAGTAGTGATTTTGTC	
uCA6	GATGCTCGCCATGGCTAACGACCCGATCACCTCT	
dCA6	CACGCGCCTCGAGCTAAGAGGTACGCAGCGGGTT	
uHAV	GGTCAAGGCCCATGGCTGTTGGTGACGACTCTGGT	
dHAV	GCGCCGCTCGAGCTAACGAGACATCATAGATTC	
uCA5	GGCCCGGGGATCCGGTGACCCGATCGCTGAC	The primer pairs uCA5/dCA5 and uPV1/dPV1 were used to amplify the VP1 of CA5 and PV1. uCA5, uPV1 contain *Bam*H I restriction sites (underlined), dCA5, dPV1 contain *Sac* I restriction sites (underlined).
dCA5	GGCCCGGGAGCTCCTAGGTGGTGGTGATAGAGGT	
uPV1	GGCGCGAGGGGATCCGGTCTGGGTCAGATGCTG	
dPV1	GCGCCGGAGCTCCTAGTAGGTGGTC AGGTCTTT	
uCB3	GCTGCTCGAAGCTTGCGGTCCGGTTGAAGACGCT	The primer pair uCB3/dCB3 was used to amplify the VP1 of CB3. uCB3 contains *Hin*d III restriction site (underlined), dCB3 contains *Xho* I restriction sites (underlined).
dCB3	CACGCGCCTCGAGCTAGAAAGCACCGGTGTTGGT	

Colour key
> 0.6
0.3 - 0.6
< 0.3

(a)

Inhibition to anti- EV71 VP1 (August 2013)

	EV71[a]	CA16[a]	CA5[a]	CA6[a]	CB3[b]	PV1[c]	HAV[d]
EV71[a]		.654**	.627**	.766**	.536**	.531**	0.027
CA16[a]			.906**	.895**	.466**	.519**	0.274
CA5[a]				.900**	.631**	.628**	0.195
CA6[a]					.643**	.683**	0.189
CB3[b]						.829**	-0.057
PV1[c]							0.124
HAV[d]							

Inhibition to anti- EV71 VP1 (May 2014)

	EV71[a]	CA16[a]	CA5[a]	CA6[a]	CB3[b]	PV1[c]	HAV[d]
EV71[a]		.693**	.677**	.627**	.929**	.707**	0.23
CA16[a]			.960**	.958**	.712**	.556**	0.209
CA5[a]				.963**	.695**	.554**	0.185
CA6[a]					.650**	.533**	0.175
CB3[b]						.811**	0.176
PV1[c]							0.195
HAV[d]							

(b)

Inhibition to anti- CA16 VP1 (August 2 13)

	EV71[a]	CA16[a]	CA5[a]	CA6[a]	CB3[b]	PV1[c]	HAV[d]
EV71[a]		0.194	0.106	.417**	-0.094	0.022	.413**
CA16[a]			.725**	.622**	.620**	.484**	-0.176
CA5[a]				.621**	.866**	.525**	-.296*
CA6[a]					.493**	.492**	-0.016
CB3[b]						.585**	-.433**
PV1[c]							-0.218
HAV[d]							

Inhibition to anti- CA16 VP1 (May 2014)

	EV71[a]	CA16[a]	CA5[a]	CA6[a]	CB3[b]	PV1[c]	HAV[d]
EV71[a]		.378**	0.259	0.286	.306*	0.226	.345*
CA16[a]			.782**	.807**	.738**	.741**	0.003
CA5[a]				.946**	.944**	.784**	0.02
CA6[a]					.941**	.843**	0.031
CB3[b]						.830**	0.057
PV1[c]							0.128
HAV[d]							

(c)

Inhibition to anti- CA5 VP1 (August 2013)

	EV71[a]	CA16[a]	CA5[a]	CA6[a]	CB3[b]	PV1[c]	HAV[d]
EV71[a]		0.217	-0.041	.314*	-0.003	-0.041	.547**
CA16[a]			.758**	.863**	.672**	.360*	-0.049
CA5[a]				.690**	.738**	.533**	-.323*
CA6[a]					.647**	.438**	-0.059
CB3[b]						.494**	-.308*
PV1[c]							-0.172
HAV[d]							

Inhibition to anti- CA5 VP1 (May 2014)

	EV71[a]	CA16[a]	CA5[a]	CA6[a]	CB3[b]	PV1[c]	HAV[d]
EV71[a]		.534**	.431**	.621**	.373**	.369**	.286*
CA16[a]			.772**	.816**	.628**	.549**	-0.114
CA5[a]				.752**	.705**	.372**	-.302*
CA6[a]					.733**	.642**	-0.157
CB3[b]						.490**	-0.261
PV1[c]							0.133
HAV[d]							

(d)

Inhibition to anti- CA6 VP1 (August 2013)

	EV71[a]	CA16[a]	CA5[a]	CA6[a]	CB3[b]	PV1[c]	HAV[d]
EV71[a]		0.173	0.235	.424**	-0.07	0.073	.474**
CA16[a]			.826**	.641**	.547**	0.242	0.234
CA5[a]				.568**	.677**	.318*	0.225
CA6[a]					.310*	.321*	0.221
CB3[b]						.506**	-0.117
PV1[c]							0.111
HAV[d]							

Inhibition to anti- CA6 VP1 (May 2014)

	EV71[a]	CA16[a]	CA5[a]	CA6[a]	CB3[b]	PV1[c]	HAV[d]
EV71[a]		.338*	.444**	0.257	0.253	0.155	.478**
CA16[a]			.726**	.716**	.681**	.442**	0.021
CA5[a]				.607**	.709**	.393**	0.127
CA6[a]					.635**	.468**	-0.017
CB3[b]						.546**	0.043
PV1[c]							0.072
HAV[d]							

(e)

Inhibition to anti- CB3 VP1 (August 2013)

	EV71[a]	CA16[a]	CA5[a]	CA6[a]	CB3[b]	PV1[c]	HAV[d]
EV71[a]		.475**	.369**	.528**	-0.043	.312*	.606**
CA16[a]			.788**	.859**	-0.021	.395**	.491**
CA5[a]				.773**	0.153	0.252	.380**
CA6[a]					-0.161	.355*	.437**
CB3[b]						-0.008	-0.088
PV1[c]							0.186
HAV[d]							

Inhibition to anti- CB3 VP1 (May 2014)

	EV71[a]	CA16[a]	CA5[a]	CA6[a]	CB3[b]	PV1[c]	HAV[d]
EV71[a]		.728**	.732**	.778**	0.138	.387**	.466**
CA16[a]			.901**	.848**	0.098	0.21	.365*
CA5[a]				.878**	0.172	0.199	.409**
CA6[a]					.340*	.399**	.434**
CB3[b]						.319*	0.162
PV1[c]							0.247
HAV[d]							

(f)

Inhibition to anti- PV1 VP1 (August 2013)

	EV71[a]	CA16[a]	CA5[a]	CA6[a]	CB3[b]	PV1[c]	HAV[d]
EV71[a]		.738**	.691**	.732**	.381**	0.141	.686**
CA16[a]			.940**	.965**	.536**	0.169	.552**
CA5[a]				.928**	.644**	0.262	.467**
CA6[a]					.550**	0.2	.524**
CB3[b]						.508**	.285*
PV1[c]							0.163
HAV[d]							

Inhibition to anti- PV1 VP1 (May 2014)

	EV71[a]	CA16[a]	CA5[a]	CA6[a]	CB3[b]	PV1[c]	HAV[d]
EV71[a]		.528**	.436**	.502**	0.212	-0.134	.479**
CA16[a]			.831**	.906**	0.21	0.012	.501**
CA5[a]				.839**	0.267	0.112	.453**
CA6[a]					.309*	0.078	.420**
CB3[b]						.427**	0.145
PV1[c]							-0.038
HAV[d]							

Figure S1. Correlation analysis of inhibition to anti-VP1 reactions of EV71, CA16, CA5, CA6, CB3, and PV1 by VP1 of EV71, CA16, CA5, CA6, CB3, PV1 and HAV in serum samples from August 2013 and May 2014. * represents $p < 0.05$. ** represents $p < 0.01$. [a]Enterovirus A. [b]Enterovirus B. [C]Enterovirus C. [d]Hepatitis A virus.

References

1 Yu, W. Z. *et al.* Poliomyelitis eradication in China: 1953-2012. *The Journal of infectious diseases* **210 Suppl 1**, S268-274, doi:10.1093/infdis/jit332 (2014).

2 Fangcheng, Z. *et al.* Era of vaccination heralds a decline in incidence of hepatitis A in high-risk groups in China. *Hepat Mon* **12**, 100-105, doi:10.5812/hepatmon.838 (2012).

3 Cui, F. *et al.* Development, production, and postmarketing surveillance of hepatitis A vaccines in China. *Journal of epidemiology / Japan Epidemiological Association* **24**, 169-177 (2014).
